# Poly(Lactic Acid)/Graphite Nanoplatelet Nanocomposite Filaments for Ligament Scaffolds

**DOI:** 10.3390/nano11112796

**Published:** 2021-10-22

**Authors:** Magda Silva, Carina Gomes, Isabel Pinho, Hugo Gonçalves, Ana C. Vale, José A. Covas, Natália M. Alves, Maria C. Paiva

**Affiliations:** 1 3B’s Research Group, I3Bs—Research Institute on Biomaterials, Biodegradables and Biomimetics, University of Minho Headquarters of the European Institute of Excellence on Tissue Engineering and Regenerative Medicine, Avepark, Barco, 4805-017 Guimarães, Portugal; magda.sof.g.silva@gmail.com (M.S.); cat.cvale@gmail.com (A.C.V.); 2 ICVS/3B’s, Associate PT Government Laboratory, 4710-057 Braga/4805-017 Guimarães, Portugal; 3 Department of Polymer Engineering, Institute for Polymers and Composites, University of Minho, 4800-058 Guimarães, Portugal; carinagomes.96@hotmail.com (C.G.); pg38948@alunos.uminho.pt (I.P.); hugosousagoncalves@protonmail.com (H.G.); jcovas@dep.uminho.pt (J.A.C.)

**Keywords:** ligament, biomedical applications, composites, 3D-printed scaffold, textile-engineered scaffold, functionalized graphene, PLA

## Abstract

The anterior cruciate ligament (ACL) is one of the most prone to injury in the human body. Due to its insufficient vascularization and low regenerative capacity, surgery is often required when it is ruptured. Most of the current tissue engineering (TE) strategies are based on scaffolds produced with fibers due to the natural ligament’s fibrous structure. In the present work, composite filaments based on poly(L-lactic acid) (PLA) reinforced with graphite nanoplatelets (PLA+EG) as received, chemically functionalized (PLA+f-EG), or functionalized and decorated with silver nanoparticles [PLA+((f-EG)+Ag)] were produced by melt mixing, ensuring good filler dispersion. These filaments were produced with diameters of 0.25 mm and 1.75 mm for textile-engineered and 3D-printed ligament scaffolds, respectively. The resulting composite filaments are thermally stable, and the incorporation of graphite increases the stiffness of the composites and decreases the electrical resistivity, as compared to PLA. None of the filaments suffered significant degradation after 27 days. The composite filaments were processed into 3D scaffolds with finely controlled dimensions and porosity by textile-engineered and additive fabrication techniques, demonstrating their potential for ligament TE applications.

## 1. Introduction

Ligaments are formed by dense collagenous tissues that connect bones, allowing body motion and assuring joint stability, and are constantly exposed to mechanical loadings [1,2]. Adult ligaments exhibit poor healing capacity and limited vascularization [2]. In particular, injuries of the anterior cruciate ligament (ACL) are common and particularly frequent in the young and physically active population [1,3], and often require surgical intervention [3]. The recurring complications of current grafts have prompted a growing interest in the development of novel materials and tissue-engineered (TE) solutions for ACL reconstruction [4].

Most current scaffolds are essentially composed of fibers [1], mimicking the architecture and the biomechanical properties of native ligament tissue [5]. The major variations between scaffolds relate to the geometrical fiber organization [6], most of them being produced by textile techniques such as braiding, twisting, or knitting [6,7,8]. The current demands for easier, faster, and customizable solutions stimulated the search for 3D printing approaches [9,10].

PLA and its derivatives are accepted as safe for humans, with several PLA-based formulations being already approved by the FDA for clinical applications such as sutures, scaffolds, cell carriers, and drug delivery systems [11]. PLA has low immunogenicity and may cause only a slight or mild reaction to the foreign body [12,13,14]. However, its mechanical response is poor, making it difficult to mimic the ligament’s properties. Thus, PLA-based hybrid composites have been widely adopted to produce fibers and fibrous scaffolds with enhanced properties [15,16].

Graphene presents outstanding mechanical, thermal, and electrical properties [17]. The use of graphene-based materials such as graphene nanoplatelets (GNPs), carbon nanotubes (CNTs), or graphite nanoflakes has been effective to reinforce PLA and other polymer matrices [18,19]. The resulting composites are expected to exhibit enhanced mechanical, electrical, and thermal properties [17,20], increasing their potential use in different biomedical applications, such as biosensing, drug delivery, and tissue engineering [21,22]. For example, Pinto et al. [18] produced nanocomposites containing PLA/COOH functionalized carbon nanotubes (CNTs-COOH) and PLA/GNPs and reported that the carbon nanostructures exhibited improved mechanical performance, approaching the range of ligament properties [18]. 

The functionalization of graphene is a good strategy to enhance its compatibility with a polymeric matrix [23]. A covalent functionalization based on 1,3-dipolar cycloaddition reaction of an azomethine ylide (DCA) has been successfully applied to graphene, preserving its inherent structure [24,25,26]. For example, CNTs were functionalized by DCA forming pyrrolidine groups at their surface that reacted with the ester groups of PLA, forming covalent bonds with the polymer. The resulting composites presented higher tensile properties and lower electrical resistivity [20].

The possibility of incorporating antibacterial components into scaffolds may ensure proper healing and postoperative regeneration of the scaffold implantation site [27,28,29]. Silver nanoparticles have shown to be particularly beneficial for tissue regeneration, not only by preventing bacterial adhesion and infection, but also by accelerating the healing process and production of extracellular matrix components [28,30,31]. Functionalized graphene surfaces can be decorated with silver nanoparticles through a reaction based on the reduction of silver ions by N,N-dimethylformamide. This decoration was successfully applied by Silva et al. [32] to amine-functionalized single-walled CNTs.

The aim of this work is the production by melt processing of composite filaments based on PLA reinforced with graphite nanoplatelets, as received, chemically functionalized, and decorated with silver nanoparticles, whilst ensuring good dispersion of the various fillers. All filaments were produced with diameters of approximately 0.25 and 1.75 mm for the subsequent preparation of textile-engineered and 3D-printed scaffolds, respectively. The thermal and mechanical properties, morphology, biodegradation, and structure of the filaments, as well as their suitability for the production of tissue engineering scaffolds by textile fabrication and additive manufacturing, were assessed.

## 2. Materials and Methods

### 2.1. Production of Functionalized Micronized Graphite

Micrograf HC11 (hereafter designated as EG), a graphite subjected to grinding and exfoliation, with a purity of 99.5%, nominal equivalent diameter of approximately 10 µm, and few tens of nanometers of thickness, was obtained from Nacional de Grafite Lda (Minas Gerais, Brazil). The functionalized EG (f-EG) was obtained by using the solvent-free 1,3-dipolar cycloaddition reaction (DCA), adapted from the procedure described for CNTs [24]. The reagents used were α-amino acid N-benzyloxycarbonylglycine (Z-gly-OH, 99%) and paraformaldehyde, both from Sigma-Aldrich, St. Louis, MO, USA, and a small amount of diethyl ether (Fisher Scientific, Loughborough, UK) to aid homogenization. The solid mixture was heated for 3 h at 250 °C. The functionalized products were washed with absolute ethanol, hexane (95% n-hexane), and acetone (all from Fisher Scientific, Loughborough, UK), and dried for 2 h at 150 °C in vacuum.

### 2.2. Anchoring of Silver Nanoparticles onto Functionalized Exfoliated Graphite

The procedure of anchoring silver nanoparticles onto f-EG was adapted from a method described for CNTs [32] consisting of the reduction reaction of silver ions (Ag+) using N,N-dimethylformamide (DMF, from Panreac, Barcelona, Spain), obtaining [(f-EG)+Ag]. A total of 140 mg of silver nitrate was mixed with 8 mL of absolute ethanol (both from Fisher Scientific, Loughborough, UK) and left under magnetic stirring, at room temperature, for 15 min. At the same time, 280 mg f-EG was mixed with 16 mL DMF and magnetically stirred, at room temperature, for 15 min. These two suspensions were then mixed together and stirred for 72 h, protected from light, being subjected to ultrasounds for 15 min every 24 h. Finally, the product was filtered and washed with diethyl ether and hexane (95% n-hexane from Fisher Scientific, Loughborough, UK) and then dried for 2 h at 150 °C under vacuum.

### 2.3. Characterization of Functionalized Graphite

Thermogravimetric analysis (TGA) was performed on Q500 equipment (TA Instruments^®^, New Castle, DE, USA). EG, f-EG, and [(f-EG)+Ag] were placed in a platinum crucible and heated from 40–800 °C at 10 °C min^−1^ under a nitrogen atmosphere of 50 mL min^‒1^.

Raman spectra were acquired using a LabRAM HR Evolution Raman spectrometer with a microscope (Horiba Scientific, Piscataway, NJ, USA) using a laser with a wavelength of 532 nm and a grating of 600 gr mm^‒1^. The results were analyzed with the Horiba Scientific’s Labspec 6 (version 6.4.4) Spectroscopy Suite Software (Horiba France SAS, Longjumeau, France) and the peak positions were determined by applying a baseline (in LabSpec 6).

Scanning electron microscopy (SEM) and energy-dispersive X-ray spectroscopy (EDS) were carried out using a FEI Nova 200 FEG-SEM/EDS (FEI Europe Company, Hillsboro, OR, USA). The samples were previously sputtered with a gold layer, using a sputter coater 108A (Cressington, Watford, UK).

### 2.4. Production of Filaments

**Figure 1:**
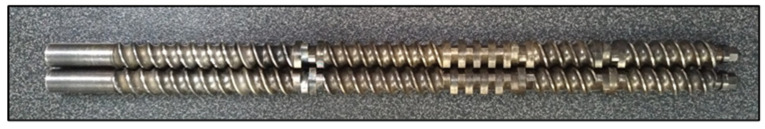
Screw profile used for the production of the composite filaments. The screws comprise three mixing zones separated by conveying elements. Polymer and fillers are fed at the conveying zone upstream. The polymer melts at the first mixing section. The remaining two mixing zones promote dispersive and distributive mixing.

**Figure 2:**
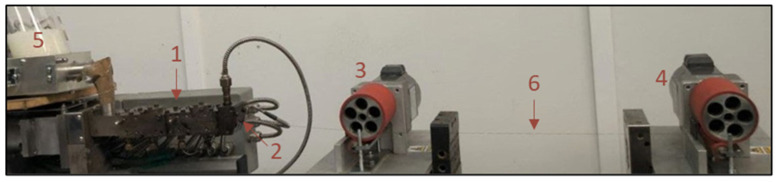
Extrusion setup: 1—Twin-screw extruder; 2—Extrusion die; 3—Drawing roll 1; 4—Drawing roll 2; 5—Feeder; 6—Filament.

PLA with a melt flow index of 3 g/10 min (Luminy LX175 from Total Corbion, Gorinchem, The Netherlands) was used as matrix of the composites. Filaments of PLA and PLA with 0.25, 0.5, 1, and 2 wt.% of EG, f-EG, and [(f-EG)+Ag] were produced with diameters of circa 0.25 mm (FilText) and 1.75 mm (Fil3D), using an intermeshing co-rotating twin-screw extruder (Rondol Microlab, Nancy, France) with a screw diameter of 10 mm and a length-to-diameter ratio (L/D) of 25 (Figure 1), coupled to an extrusion rod die with 2 mm of diameter and two pulling rolls (see Figure 2). The screws comprised three mixing zones separated by conveying elements. The polymer was fed upstream by a volumetric feeder (Piovan MDP1, S. Maria di Sala, VE, Italy) at the rate of 2.81 g·min^−1^ or 0.62 g·min^−1^ for the production of Fil3D and FilText, respectively. The fillers were added manually at the same location, at a rate adjusted according to the desired concentration. The speed of the puling rolls was adjusted to obtain filament diameters of 1.75 and 0.25 mm. The operating parameters used for the production of the filaments Fil3D and FilText are presented in Supplementary Materials Table S1. The PLA was dried for 8 h at 70 °C and 3 h at 100 °C before processing.

### 2.5. Characterization of the Composite Filaments

The filaments were analyzed on a LabRAM HR Evolution Raman spectrometer (Horiba Scientific, Piscataway, NJ, USA) equipped with a laser with wavelength of 532 nm and a grating of 600 gr mm^−1^. The results were analyzed with the LabSpec6 (version 6.4.4) software.

TGA analysis was carried out on Q500 equipment (TA Instruments^®^, New Castle, DE, USA). The samples were placed in a platinum crucible and heated from 40–800 °C at 10 °C min^−1^ under a nitrogen atmosphere of 50 mL min^−1^.

The filaments’ cross-sections were analyzed by SEM, using a FEI Nova 200 FEG-SEM/EDS (FEI Europe Company, Hillsboro, OR, USA). Composite filaments with [(f-EG)+Ag] were also analyzed by EDS, using the same equipment.

Cross-sections with 3 µm thickness of each type of filament were cut with a Leica EM UC6 ultramicrotome and placed over a glass coverslip with Canada balm. Due to the reduced diameter, FilText filaments were embedded in epoxy to facilitate sample microtoming. Then, the cross-sections were analyzed on an Olympus BH-2 optical microscope using a 40× objective, in transmission mode. The images obtained from OM were then analyzed with the ImageJ software for statistical analysis.

Differential scanning calorimetry (DSC) measurements were performed on a DSC 200 F3 Maia (Netzsch-Gerätebau GmbH, Selb, Germany) under a constant flow of nitrogen. The samples were heated from 30–190 °C at 5 °C min^−1^, cooled and then reheated up to 190 °C, at the same rate. The results were analyzed using the Netzsch Proteus software. The degree of crystallinity (χ_c_) of PLA and composite filaments was calculated by:

(1) $$\chi_{c}\;\left(\%\right)=\frac{\Delta H_{m}}{\varphi_{PLA}\times \Delta H_{m}{}^{0}}\times 100$$

where ∆*H_m_* is the enthalpy of fusion (J g^−1^), φ*_PLA_* is the weight fraction of PLA in the composites, and ∆*H_m_*^0^ is the enthalpy of PLA for 100% crystallinity, considered equal to 93.7 J g^−1^ [33].

Dynamic mechanical analysis (DMA) tests were carried out using Tritec 2000 B equipment (Triton Technology, Grantham, UK), equipped with the tensile mode and a grip distance of 15 mm. Filament samples were cut with a length of approximately 30 mm. The diameter of each sample was measured on three different places along the filament length, using a micrometer (Mitutoyo, Kawasaki, Japan). The tests were carried out between 10 °C and 70 °C, with a step of 2 °C. A static pre-load of 1 N was used and the measurements were made at a frequency of 1 Hz, which corresponds to the physiological loading frequency defined as an ASTM standard frequency to determine T_g_ (ASTM E1640–07) [34]. At least three specimens were tested for each composition.

The electrical resistivity of the composite filaments was measured on a Keithley SMU 2635B SourceMeter^®^ (Keithley Instruments Inc., Cleveland, OH, USA). The test specimens were cut with 20 mm length and their diameter was measured on three different places along the filament length, using a micrometer (Mitutoyo, Kawasaki, Japan). Each sample was clamped by the electrodes and the current across the test specimen was measured with the application of a 10 V potential.

To assess the biodegradation, composite filaments with 0.25 and 0.5 wt.% of fillers, previously dried and weighed, were immersed in phosphate-buffered saline (PBS) and stored in an incubator at 37 °C for 7, 14, 21, and 28 days. Every two days the PBS solutions were replaced by fresh solutions; the filaments were removed from the PBS, washed with distilled water, dried, and weighed. The weight loss was calculated by:

(2) $$Weight\;loss\;\left(\%\right)=\frac{m_{i}-m_{f}}{m_{i}}\times 100$$

where *m_i_* is the weight of the filament before immersion in PBS and *m_f_* is the dry weight of the filament at each time. Each experiment was repeated three times.

### 2.6. Scaffold Production and Characterization

Braided and 3D-printed scaffolds were manufactured using FilText and Fil3D reinforced with 0.5 and 2 wt.% of fillers to evaluate their potential usefulness for scaffold production.

Three-dimensional-printed scaffolds were designed using the Ultimate Cura software and produced by an Ender-3 3D Printer adopting the following parameters: infill density of 50%, infill linear pattern (0 and 90 °C), and a layer height of 0.15 mm. The build platform was set to 80 °C, the nozzle temperature to 185 °C, and the printing speed to 45 mm·s^−1^. The textile-engineered scaffold was produced through a kumihimo hand braiding technique, with a circular stand. The scaffold is formed by an exterior braided structure formed by 8 FilText and an inner part containing 4 pairs of bundles. The bundles were aligned in parallel and tied together with a suture of the same material and each was formed by 8 braided FilText. Both scaffolds exhibit approximately 32 mm of length and 9 mm of diameter, similar to the dimensions of the native ACL.

The scaffolds were analyzed using the Digital Microscope Leica DMS1000 in order to identify their morphology and qualitatively estimate the shape, size, and distribution of the pores. Images were collected with a magnification of 1.6× *g*.

## 3. Results and Discussion

### 3.1. Functionalization of Graphite

**Figure 3:**
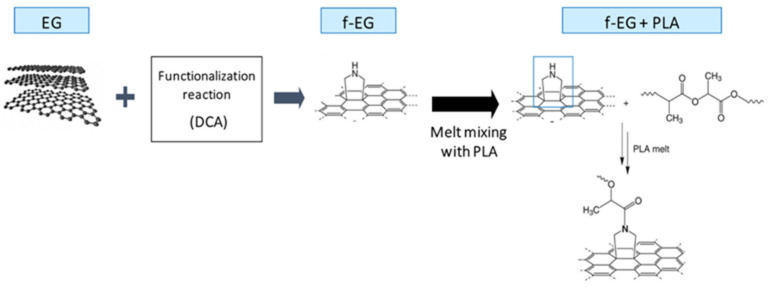
Schematic representation of the EG functionalization by the DCA reaction, obtaining f-EG as well as its further interaction with PLA under melt processing conditions.

The functionalization of EG to form f-EG was carried out using a DCA reaction of an azomethine ylide. This reaction is expected to functionalize the EG surface by covalent bonding pyrrolidine (cyclic amine) groups without structural damage to the EG [24]. The cyclic amine may react with PLA under melt processing conditions, establishing a strong interface that enhances stress transfer from the polymer to the reinforcement [20]. This process is represented in Figure 3.

**Figure 4:**
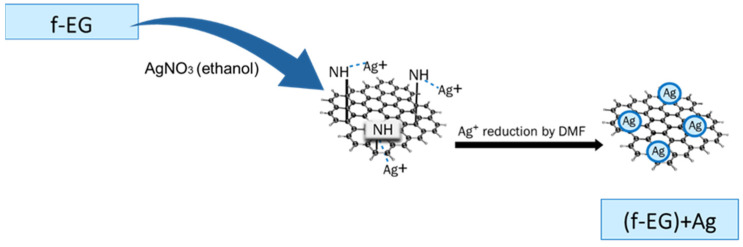
Schematic representation of the decoration of f-EG with silver nanoparticles, obtaining [(f-EG)+Ag].

Silver nanoparticles were anchored onto f-EG through a reaction based on the reduction of silver ions by DMF [32]. At the end of this step, silver decorated f-EG [(f-EG)+Ag] was obtained, as represented in Figure 4.

### 3.2. Characterization of Functionalized Graphite

#### 3.2.1. Thermogravimetry

**Figure 5:**
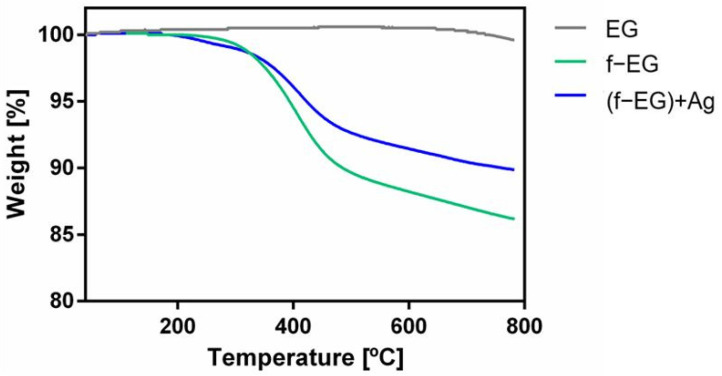
TGA curves for pristine EG, f-EG, and [(f-EG)+Ag].

The effect of functionalization on the thermal stability of graphite was assessed by TGA, performed on EG, f-EG, and [(f-EG)+Ag]. The results are presented in Figure 5.

The TGA curves of f-EG and [(f-EG)+Ag] exhibit a similar shape. The beginning of thermal degradation is observed at a lower temperature for [(f-EG)+Ag], but the weight loss at 800 °C is lower than that observed for f-EG. As shown by Silva et al. [23], pristine EG is thermally stable in the analyzed temperature range, as expected for pristine materials with a low contamination level. Thus, the weight loss observed is due to the thermal degradation of the organic moieties bonded to EG through the DCA reaction. Since [(f-EG)+Ag] was expected to have the same organic groups that were bonded in f-EG, the weight loss difference between f-EG and [(f-EG)+Ag] results from the silver nanoparticle residue that remains stable within the temperature range of the TGA tests. This indicates a successful functionalization of EG and addition of Ag nanoparticles. The weight loss of EG, f-EG, and [(f-EG)+Ag] was 0.4, 13.8, and 10.1, respectively. The functionalization yield was 13.4 wt.% and silver nanoparticle content was 3.7 wt.%.

#### 3.2.2. Raman Spectroscopy

**Figure 6:**
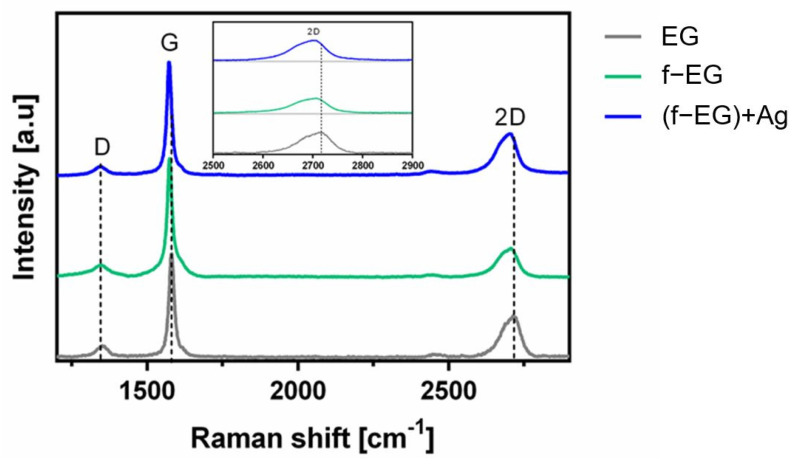
Raman spectra of EG, f-EG, and [(f-EG)+Ag].

The Raman spectra of graphite and graphene derivatives typically exhibit three characteristic bands designated by D, G, and 2D (Figure 6) [35]. The D band, located at 1350 cm^−1^, indicates the presence of sp^3^ carbon atoms, demonstrating the existence of defects in the sp^2^ hybridized carbon lattice. The G band, near 1580 cm^−1^, is due to in-plane vibration of the ordered sp^2^ bonded carbon atoms [36,37]. The normalized Raman spectra of EG, f-EG, and [(f-EG)+Ag] allowed the measurement of the intensity ratios of D and G bands (I_D_/I_G_).

The I_D_/I_G_ ratio is 0.24, 0.10, and 0.13 for EG, f-EG, and [(f-EG)+Ag], respectively. The low I_D_/I_G_ ratio is indicative of few defects in the pristine graphite structure. Moreover, the I_D_/I_G_ ratio decreased from 0.24 to 0.10 after functionalization, indicating fewer defects present in the f-EG compared to the pristine EG. This may result from the functionalization method, consisting of the cycloaddition reaction to the graphite C–C double bonds, which may lead to the selection of the less defective graphite flakes [38]. Additionally, although sp^3^ carbon is generated by functionalization, the reaction does not damage the graphene structure, keeping the hexagonal lattice. Finally, the EG material remaining with a low degree of functionalization, or not functionalized, may be separated during the washing and sonication procedures, leaving mostly the functionalized material (f-EG). With the addition of silver nanoparticles, the I_D_/I_G_ intensity ratio between f-EG and [(f-EG)+Ag] was maintained or slightly increased, which may indicate incipient disturbance of the in-plane sp^2^ carbon lattice [23] due to the addition of Ag nanoparticles to the graphene layers. Nevertheless, this is a negligible variation.

The 2D band observed near 2700 cm^−1^ correlates with the quality of graphene and with the number of layers of graphene by the shape, width, and position of the peak [35]. This band is at double the frequency of the D band [39]. As Zhu et al. [35] and Ferrari et al. [40] reported in their studies, with an increasing number of graphene layers the 2D peak moves to higher wavenumbers and becomes broader, while pure graphene exhibits a single sharp 2D peak with higher intensity relative to the G peak [40]. In Figure 6, a shift of the 2D peak wavenumber is observed, decreasing from 2717 cm^−1^, for EG, to 2706 and 2698 cm^−1^, for f-EG and [(f-EG)+Ag], respectively. This observation is consistent with the selective functionalization of the thinner and structurally more perfect pristine EG flakes. Besides, the deposition of silver nanoparticles on functionalized graphite also shifted the 2D band towards lower wavenumbers, which may suggest a charge-transfer process and chemical interaction between the Ag nanoparticles and the graphene surface after the deposition process, as reported in previous works [41].

#### 3.2.3. Scanning Electron Microscopy

**Figure 7:**
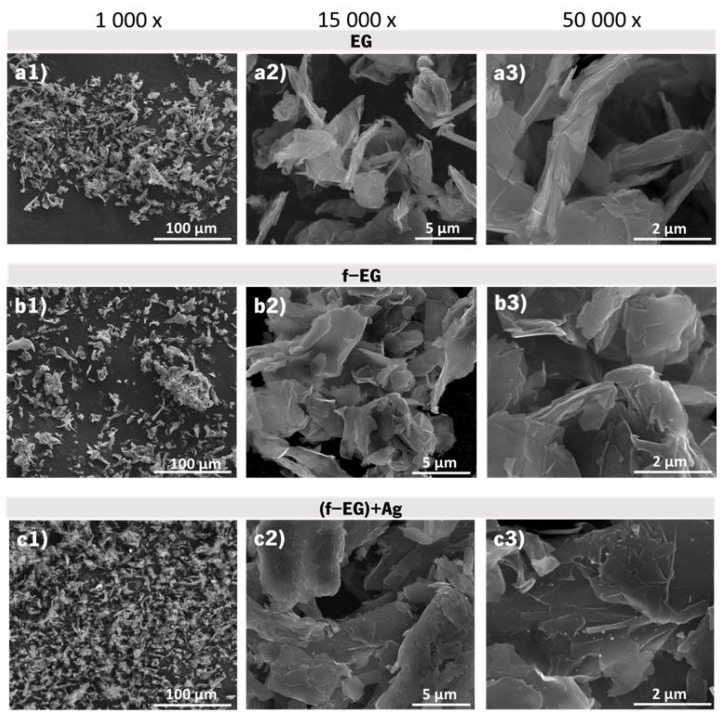
SEM images for pristine EG at different magnifications: (**a1**–**a3**); f-EG: (**b1**–**b3**) and [(f-EG)+Ag]: (**c1**–**c3**).

The graphite morphology was characterized by SEM, the images of (a) pristine EG, (b) f-EG, and (c) [(f-EG)+Ag] being displayed in Figure 7, evidencing that the graphite morphology was maintained after functionalization. EDS tests were performed for [(f-EG)+Ag] and are presented in Supplementary Materials Figure S1, showing the presence of Ag on the graphite surface.

### 3.3. Characterization of the Composite Filaments

#### 3.3.1. Macroscopic Characterization

**Figure 8:**
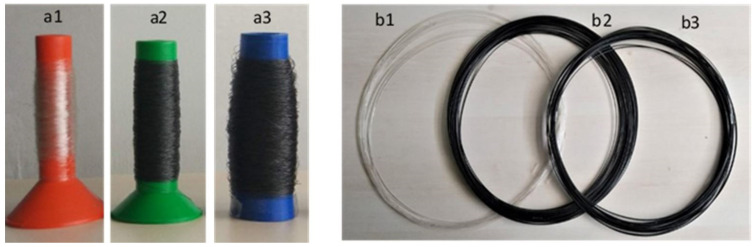
Composite filaments: FilText (**a1**—PLA; **a2**—PLA+0.5[(f-EG)+Ag]; **a3**—PLA+2[(f-EG)+Ag]) and Fil3D (**b1**—PLA; **b2**—PLA+0.5[(f-EG)+Ag]; **b3**—PLA+2[(f-EG)+Ag]).

Figure 8 presents the filaments produced by melt mixing, namely of PLA, PLA+0.5[(f-EG)+Ag], and PLA+2[(f-EG)+Ag] of FilText (Figure 8a1–a3, respectively) with an average diameter of 0.26 ± 0.03 mm and Fil3D (Figure 8b1–b3, respectively), with an average diameter of 1.71 ± 0.07 mm. All filaments exhibited good filler dispersion and a flexibility suited to the intended application. Their thermal, mechanical, and electrical properties are presented and discussed below.

#### 3.3.2. Thermogravimetric Analysis

**Figure 9:**
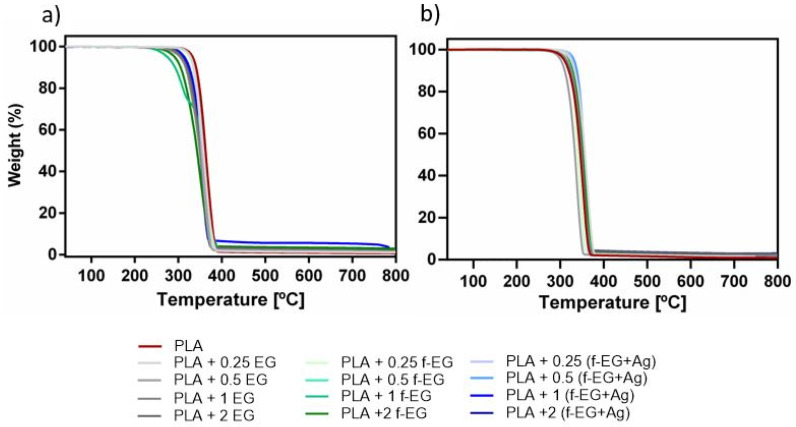
TGA thermograms of (**a**) Fil3D and (**b**) FilText.

The thermal stability of Fil3D and FilText as well as their nanoparticle weight composition were assessed by TGA. Thermal stability is an important factor due to its impact on melt processing, as well as on the end-use applications. The analysis of the thermograms of Fil3D and FilText presented in Figure 9a,b shows a single step degradation for all the compositions in the range of 300–400 °C, which occurs due to the decomposition of the PLA and functional organic groups of f-EG and [(f-EG)+Ag].

Above 400 °C the weight loss stabilizes, reaching a plateau and showing a higher residual weight of the composite filaments as compared to the PLA filament. Graphene-based materials are known for their high thermal stability under inert atmosphere, thus allowing the estimation of the filler composition by residual weight analysis. Table 1 presents the temperature at the onset of thermal degradation, as well as the residual weight measured at 800 °C. It is observed that PLA and composites present similar thermal stability, in agreement with results reported before by Paiva et al. [24] for composites with PLA and CNTs.

The residual weight percent increases with increasing reinforcement concentration and is within the nominal range, except for the composition of PLA+2EG for Fil3D and PLA+2[(f-EG)+Ag] for FilText, where the effective composition is considerably lower than the nominal value. More than five TGA tests were carried out for each composition, showing a significant variation in the final residue values. This variation is possibly due to the manual feeding of the extruder during composite preparation by melt mixing.

#### 3.3.3. Scanning Electron Microscopy

**Figure 10:**
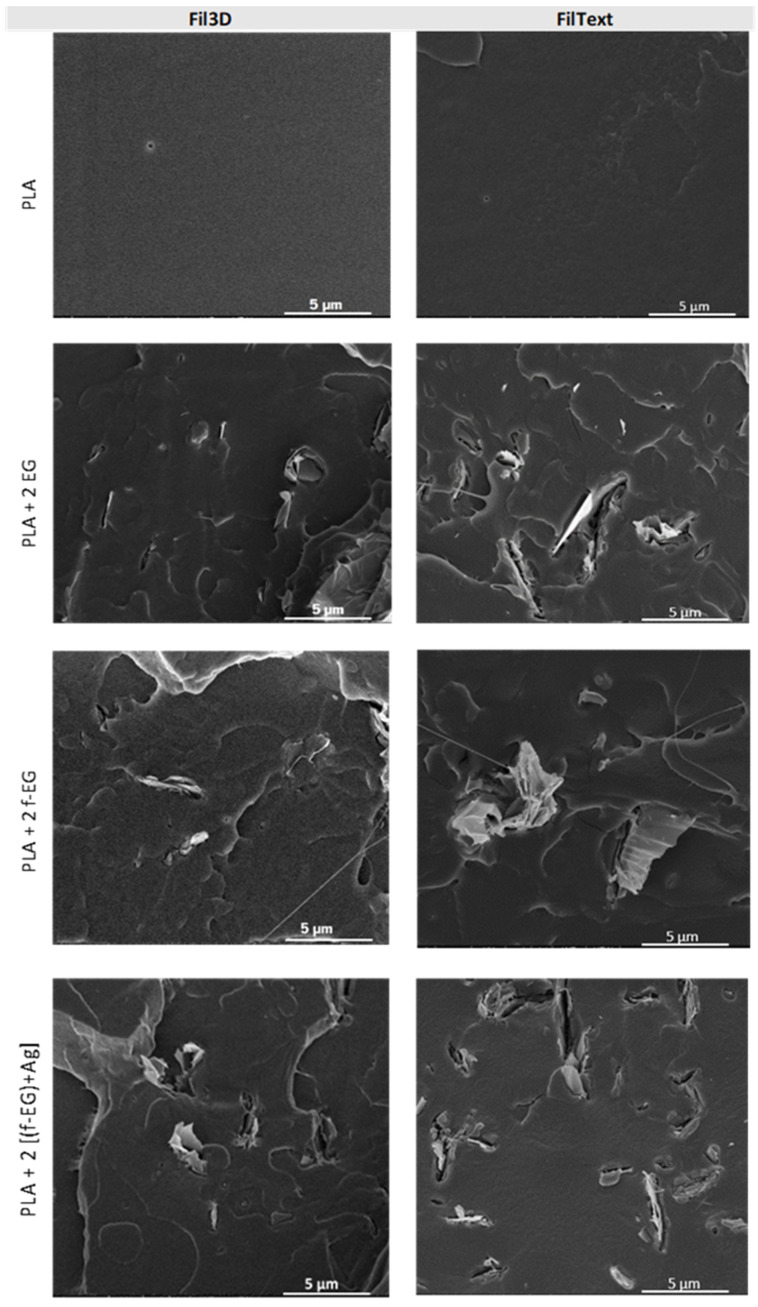
SEM images of Fil3D and FilText: PLA; PLA+2 EG; PLA+2 f-EG; PLA+2 [(f-EG)+Ag].

The morphology of all filaments and the dispersion of the reinforcement in the polymeric matrix were analyzed by SEM. The images of the cross-sections of composite filaments reinforced with 2wt.% of fillers are presented in Figure 10 and are complemented in the Supplementary Materials Figures S2 and S3. A good dispersion of nanoparticles across the composite and a good interface between PLA and graphite are observed, especially for the smaller particles. The incorporation of Ag nanoparticles does not significantly affect the filament morphology. SEM images highlighting the presence of silver nanoparticles and EDS analysis confirming their presence in Fil3D and FilText reinforced with [(f-EG)+Ag] are shown in the Supplementary Materials Figure S4.

#### 3.3.4. Optical Microscopy

**Figure 11:**
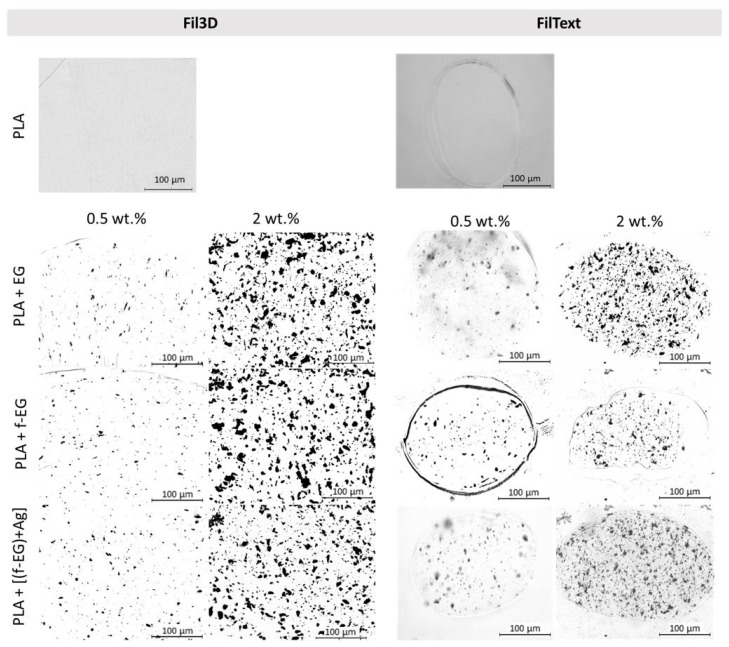
Optical microscopy images of the filaments’ cross-section, namely PLA and PLA reinforced with 0.5 wt.% and 2 wt.% of EG, f-EG, and [(f-EG)+Ag].

Figure 11 shows the OM images of the composite filament cross-sections containing 0.5 and 2 wt.% of fillers. Images of the composite filament cross-sections at all the compositions of EG, f-EG, and [(f-EG)+Ag] are displayed in the Supplementary Materials Figures S5 and S6. The statistical analysis of the average particle size, as well as the number of particles per unit composite area, measured for the composite filaments, is presented in Table 2. The average agglomerate size is slightly higher for the composites with 1 and 2 wt.% of reinforcement. Generally, filaments with functionalized graphite present a smaller number of agglomerates, except for Fil3D with 0.5 wt.% f-EG and 0.25 wt.% [(f-EG)+Ag]. However, they also present slightly larger particles, which may result from higher nanoparticle cohesion after functionalization, as observed in SEM images for f-EG. In general, the average agglomerate size tends to be smaller for FilTex filaments, which is a consequence of the higher draw ratio applied during filament production, inducing the alignment of the EG flakes along the filament length, thus showing mainly the thinner flake side on the filament cross-sections. A rough estimate for the lateral size and thickness of one flake, considering a circular flake with 10 µm diameter and 30 nm thickness, is 80 µm^2^ and 0.3 µm^2^, respectively. The average agglomerate areas obtained (Table 2) are much lower than the flat surface area of an average EG flake, which is indicative of considerable flake alignment along the filament axis, in particular for FilTex, and good EG dispersion.

#### 3.3.5. Raman Spectroscopy

**Figure 12:**
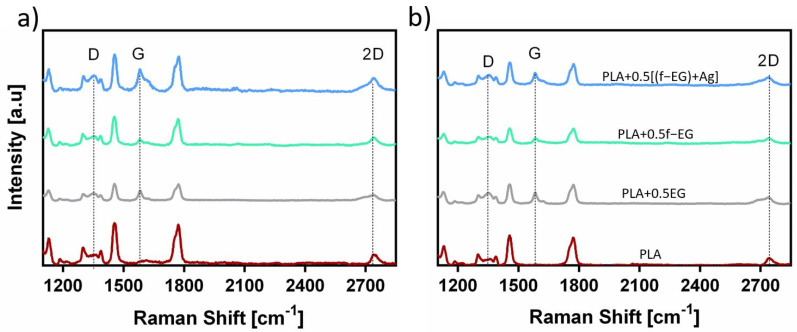
Raman spectra obtained for (**a**) Fil3D and (**b**) FilText with 0.5wt.% of EG, f-EG, and [(f-EG)+Ag].

Raman spectroscopy was performed on the filaments to further observe the graphite nanoparticles in the composite filaments. As mentioned before, all carbon-based materials show characteristic bands at a specific wavenumber in the Raman spectrum, namely D, G, and 2D bands [37]. All Raman spectra of Fil3D and FilText are similar and exhibited these three characteristics bands. Figure 12 presents the Raman spectra of filaments reinforced with 0.5 wt.% of EG, f-EG, and [(f-EG)+Ag]. PLA also presents prominent bands in the Raman spectrum, thus the wavenumbers of the D, G, and 2D bands of EG, f-EG, and [(f-EG)+Ag] were highlighted in the spectra. PLA does not show scattering in the region of the G band, and thus the corresponding wavenumber may be used to monitor the presence of graphite in composite filaments.

#### 3.3.6. Differential Scanning Calorimetry

**Figure 13:**
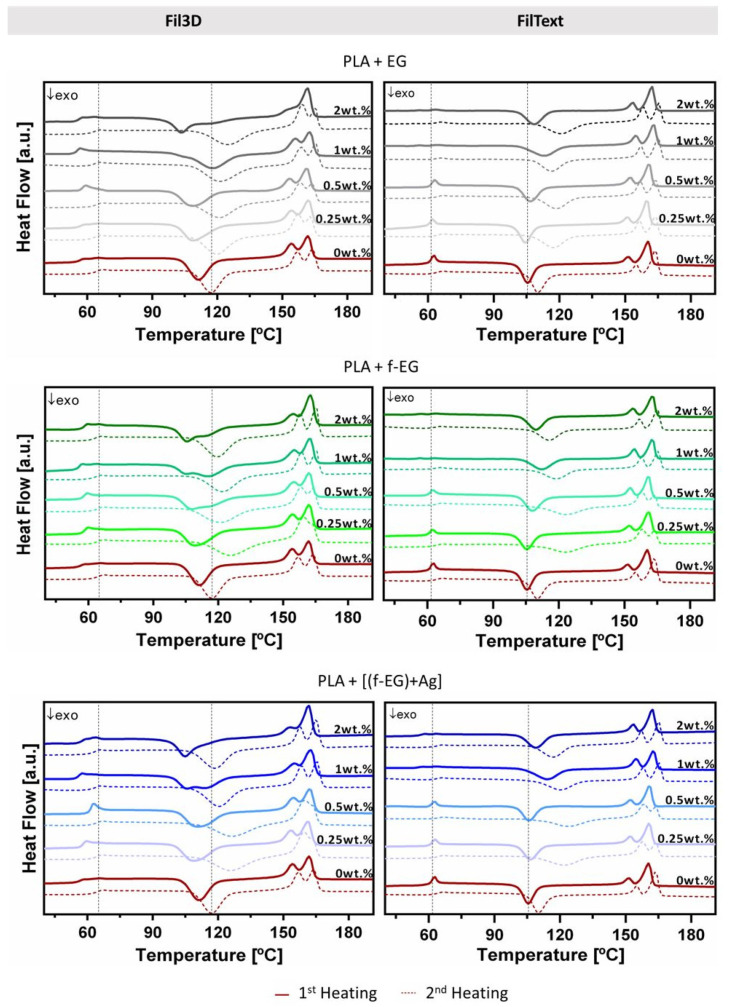
DSC scans of Fil3D and FilText, namely first and second heating, represented by continuous and dashed lines, respectively. Black dashed vertical lines mark the T_g_ and T_c_ of PLA during the second heating scan of Fil3D and first heating scan of FilText.

The analysis of the DSC results allows the characterization of the thermal behavior of PLA and the influence of filler addition. The relevant thermal characteristics (glass transition temperature, T_g_, cold crystallization temperature, T_c_, melting temperature, T_m_, melting enthalpy, ΔH_m_, cold crystallization enthalpy, ΔH_c_, and degree of crystallinity, χ_c_) of each composition obtained for the first and second heating scans are reported in Supplementary Materials Tables S2 and S3, respectively. Since the Fil3D will be used in additive manufacturing to produce 3D-printed scaffolds involving filament melting, the analysis of the second heating scan has particular interest. It can be seen in Figure 13 that the T_g_ of the composites for the second heating does not vary significantly compared to PLA and it is approximately 60 °C. Additionally, all compositions exhibited a similar T_m_ of approximately 158 °C and the double-melting peak, as was observed in PLA composites by other authors [42,43]. Conversely, FilText filaments are used as-produced to manufacture textile-based scaffolds without further heating and thus the analysis of the first heating scan is more relevant. For FilText, the T_g_ of the first and second heating is approximately 58.3 °C and 58.6 °C, respectively, and it is not significantly affected by the presence of reinforcement. All FilText filaments presented a similar T_m_ of approximately 160 °C and a double-melting peak. The cold crystallization temperature measured on the second heating scan is shifted to a higher temperature, presenting lower values for the PLA filaments compared to the composite filaments, either Fil3D and FilText. This observation suggests that PLA crystallization is delayed by the presence of the graphite nanoparticles, with and without functionalization, as reported in previous works [33]. Filaments exhibited low crystallinity, slightly increasing in the second heating with the incorporation of graphite, as was previously reported for PLA composites [44].

#### 3.3.7. Mechanical Characterization

**Figure 14:**
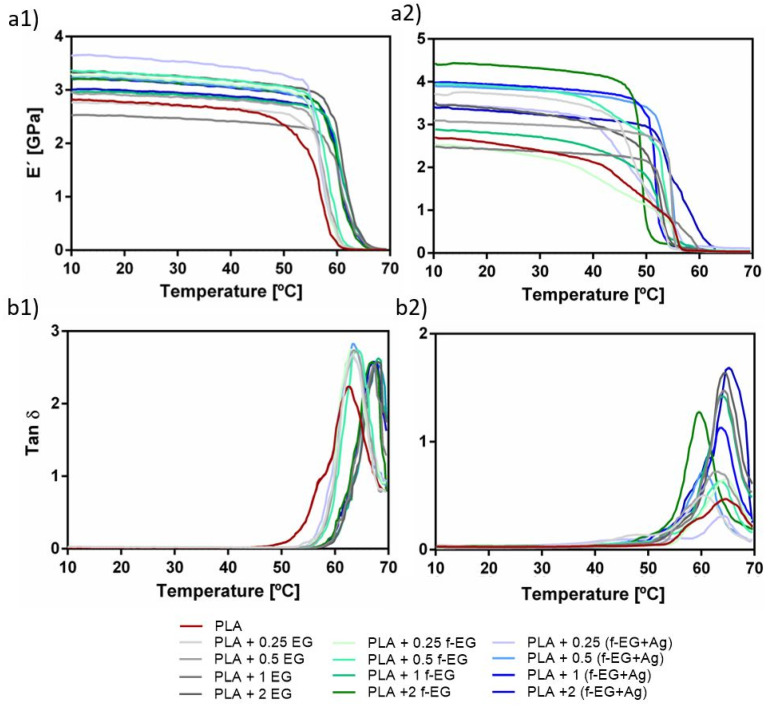
DMA spectra for the E’ of (**a1**) Fil3D and (**a2**) FilText, and tan σ of (**b1**) Fil3D and (**b2**) FilText, as a function of the temperature, ranging from 10–70 °C.

DMA was used to evaluate the effect of the incorporation of EG, f-EG, and [(f-EG)+Ag] on the mechanical and viscoelastic properties of the filaments. Figure 14 shows the DMA results obtained as a function of temperature, at 1Hz (physiological frequency), with (a1) and (a2) representing the storage modulus of Fil3D and FilText, respectively, and (b1) and (b2) the loss factor of Fil3D and FilText, respectively. 

As the temperature increases, all compositions show a gradual decrease in the storage modulus (E’), followed by a drop when T_g_ is reached. The drop in modulus is related to the material transition from the glassy to the rubbery state [45]. As expected, the composite filaments present higher E’ values compared to PLA [33], and may indicate good interfacial properties allowing for stress transfer at low deformations [45].

The loss factor, or tan δ, expressed as the ratio of the loss modulus to the storage modulus, is a measure of energy loss and provides information about the damping properties of the composites [46]. In Figure 14b1,b2, the tan δ peak observed at about 63–68 °C is related to the T_g_ of the nanocomposites. Except for PLA+0.25[(f-EG)+Ag] of FilText, all composite filaments exhibited higher tan δ values compared to PLA, indicating that they have a higher capacity to dissipate energy and damping. The viscoelastic character of these filaments has particular relevance for the application, since ligaments also exhibit viscoelastic behavior [47].

**Figure 15:**
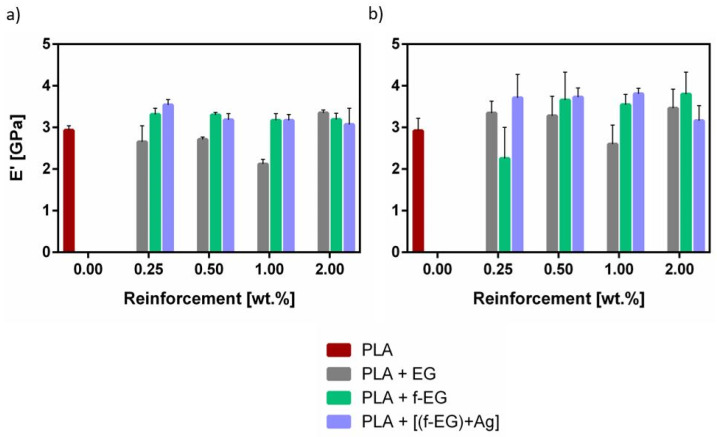
Storage modulus (E’) at 37 °C of (**a**) Fil3D and (**b**) FilText.

The analysis of E’ at body temperature is presented in Figure 15, showing that most of the composite filaments present higher E’ compared to PLA.

#### 3.3.8. Electrical Resistivity

The electrical properties of materials for TE applications are quite relevant, considering their influence on cell adhesion and growth [48]. The application of electrical fields (static and pulsing) has been widely used in orthopedic practices, namely to improve tendon [49] and ligament [50] healing and repair. ACL fibroblasts demonstrated enhanced migration speed and perpendicular alignment to the applied electric fields [50].

**Figure 16:**
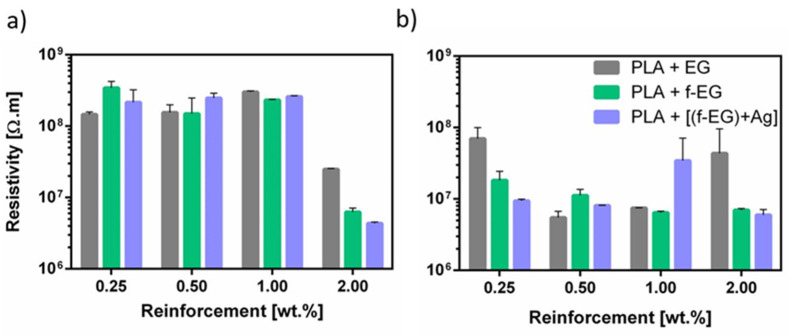
Electrical resistivity of (**a**) Fil3D and (**b**) FilText, as a function of the reinforcement.

The homogeneous dispersion of the electrically conductive graphite nanoparticles in PLA is expected to decrease the electrical resistivity of the composite [51]. Figure 16 shows the electrical resistivity of the composites as a function of the graphite content for (a) Fil3D and (b) FilText. The electrical resistivity of PLA is in the order of 10^12^ Ω·m, as reported in the literature [33,52]. The electrical resistivity of the composite filaments was determined by measuring the current after applying a voltage of 10 V. It is observed that the electrical resistivity decreases with the incorporation of EG, f-EG, and [(f-EG)+Ag], as reported in previous studies [20,51,53]. Although the composite nanoparticle concentrations are far from the electrical percolation threshold, a decrease in electrical resistivity of four orders of magnitude for Fil3D and five orders of magnitude for FilText filaments is observed. The thinner filaments present lower electrical resistivity for all graphite concentrations except for the lower concentration of pristine EG, showing that the morphology of the nanoparticle distribution was affected by the drawing conditions. The presence of graphite nanoparticles in the composite filaments, even far from the electrical percolation level, may provide a positive effect on the cellular response by allowing localized electron mobility, as reported in previous works [54,55,56].

#### 3.3.9. Biodegradation

PLA and all the composite filaments were immersed in a PBS solution at 37 °C, mimicking natural body fluids, for 7, 14, 21, and 28 days. The obtained results are detailed in Supplementary Materials Table S4. PLA did not exhibit any degradation after approximately one month in PBS, and the addition of carbon nanoparticles did not affect its degradation behavior, which is an important feature for the intended application due to the ACL’s poor healing capacity and long recovery periods [57], as it takes at least 6–9 months for complete regeneration [58,59,60].

### 3.4. Scaffold Production and Characterization

The scaffolds were produced to demonstrate the processability of the composite filaments FilText and Fil3D, their detailed characterization being the focus of future work.

Braided and 3D-printed scaffolds were manufactured using FilText and Fil3D filaments, respectively, as described above. The production of 3D-printed scaffolds was faster, easier, and more reproducible compared to braided scaffolds. While the operating conditions for 3D printing were similar for both PLA and its composites, obtaining braided scaffolds was lengthier and more difficult for PLA braids, which lacked the flexibility of composite FilText.

**Figure 17:**
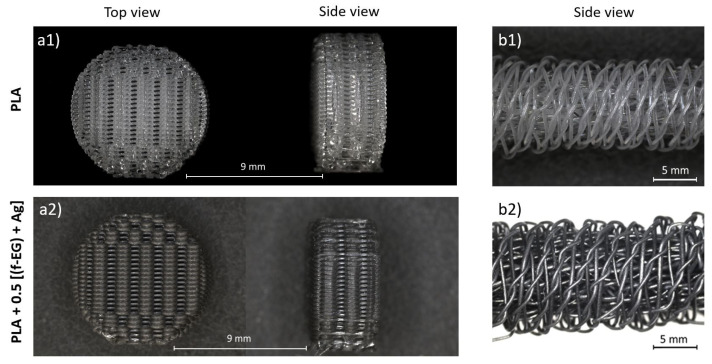
Stereoscopic magnifying glass images of PLA scaffolds obtained by (**a1**) 3D printing and (**b1**) textile engineering and of PLA+0.5[(f-EG)+Ag] scaffolds obtained by (**a2**) 3D printing and (**b2**) textile engineering.

Figure 17 illustrates the scaffolds produced by 3D printing and textile engineering, using PLA and PLA+0.5[(f-EG)+Ag] filaments. Observation with a digital microscope shows their porous structure and suitable shape. The composite scaffolds presented a morphology and pore size similar to those of neat PLA scaffolds. A preliminary assessment indicates a porosity greater than 60% for all scaffold compositions, which is appropriate for the intended application.

## 4. Conclusions

Composite filaments based on PLA reinforced with EG, f-EG, and [(f-EG)+Ag] were produced by melt processing with diameters of 0.25 and 1.75 mm for the preparation of textile-engineered and 3D-printed scaffolds for ligament application, respectively. All filaments exhibited a good dispersion of the fillers and interaction with the polymeric matrix. The filaments were thermally stable up to 130 °C in the presence of EG and functionalized EG. In general, the storage modulus of the composite filaments is approximately 3 GPa or greater at 37 °C, with tan δ values higher than those observed for PLA filaments, indicating that the addition of functionalized graphite increases the stiffness of the composites and provides a higher capacity to dissipate energy and damping. The incorporation of fillers led to a decrease in the electrical resistivity relative to neat PLA up to five orders of magnitude, with the composites with 2 wt.% of reinforcement presenting the lowest values. The degradation rate of PLA and composite filaments is low, with no significant degradation being observed after 27 days in PBS. Thus, composite filaments based on PLA and thin graphite flakes, functionalized for enhanced interface with PLA and for anchoring a small concentration of Ag as an anti-microbial agent, were produced, presenting good mechanical performance and thermal properties. The composite filaments were successfully processed into three-dimensional scaffolds with finely controlled dimensions using textile-engineered and additive fabrication techniques, demonstrating their potential for ligament TE applications.

## Figures and Tables

**Table 1:** Initial degradation temperatures of composite filaments.

Filament		Fil3D		FilText	
		T_onset_ (°C)	Residue (wt.%)	T_onset_ (°C)	Residue (wt.%)
PLA		351	-	301.6	-
PLA+0.25	EG	347	0.43 ± 0.21	318	0.20 ± 0.25
PLA+0.5		342	0.74 ± 0.22	295	0.39 ± 0.31
PLA+1		342	1.02 ± 0.88	307	1.14 ± 1.30
PLA+2		328	1.44 ± 0.37	301	2.34 ± 1.74
PLA+0.25	f-EG	342	0.23 ± 0.25	310	0.48 ± 0.74
PLA+0.5		339	0.73 ± 0.16	310	0.36 ± 0.58
PLA+1		342	1.13 ± 1.17	307	1.15 ± 0.26
PLA+2		328	2.00 ± 0.96	312	2.18 ± 1.72
PLA+0.25	(f-EG)+Ag	343	0.26 ± 0.14	323	0.21 ± 0.85
PLA+0.5		344	0.55 ± 0.27	327	0.53 ± 0.44
PLA+1		330	1.43 ± 1.40	309	1.06 ± 1.10
PLA+2		338	2.04 ± 0.85	304	1.02 ± 0.22

**Table 2:** Characterization of Fil3D and FilText’s cross-section by optical microscopy.

Filament		Average Agglomerate Size		Number of Agglomerates	
		(µm^2^)		(mm^−2^)	
		Fil3D	FilText	Fil3D	FilText
PLA+0.25	EG	2.97	1.37	2757	11,025
PLA+0.5		2.34	0.72	6691	11,675
PLA+1		5.90	7.35	6032	27,244
PLA+2		6.53	4.92	6223	29,534
PLA+0.25	f-EG	5.30	3.52	2390	6523
PLA+0.5		2.67	1.83	7735	7806
PLA+1		6.95	8.76	3378	12,045
PLA+2		10.91	3.64	4378	14,813
PLA+0.25	(f-EG)+Ag	2.53	3.38	6671	9132
PLA+0.5		4.06	0.60	5154	9749
PLA+1		11.83	3.66	2992	12,170
PLA+2		6.69	4.66	6118	20,430

## Data Availability

The data presented in this study are available on request from the corresponding author.

## Supplementary Materials

The following are available online at https://www.mdpi.com/article/10.3390/nano11112796/s1. Figure S1: SEM image and EDS analysis of [(f-EG)+Ag], Figure S2: SEM images of Fil3D: PLA; PLA reinforced with 0.25, 0.5, 1, and 2 wt.% of EG, f-EG, and [(f-EG)+Ag], Figure S3: SEM images of FilText: PLA; PLA reinforced with 0.25, 0.5, 1, and 2 wt.% of EG, f-EG, and [(f-EG)+Ag], Figure S4: SEM images and EDS analysis of Fil3D and FilText reinforced with 1 and 2 wt.% of [(f-EG)+Ag], Figure S5: Optical microscopy images of the Fil3D’s cross-section, namely PLA; PLA reinforced with 0.25, 0.5, 1, and 2 wt.% of EG, f-EG, and [(f-EG)+Ag], Figure S6: Optical microscopy images of the FilText’s cross-section, namely PLA; PLA reinforced with 0.25, 0.5, 1, and 2 wt.% of EG, f-EG, and [(f-EG)+Ag], Table S1: Operating parameters used for the production of composite filaments, Table S2: Summary of the thermal properties of Fil3D and FilText obtained for the 1st heating, Table S3: Summary of the thermal properties of Fil3D and FilText obtained for the 2nd heating, Table S4: Weight loss (%) of Fil3D and FilText.
