# Supplementary material for: Poly(Lactic Acid)/Graphite Nanoplatelet Nanocomposite Filaments for Ligament Scaffolds
Source: Nanomaterials (Basel). 2021 Oct 22;11(11):2796. doi: 10.3390/nano11112796 (PMC8625229; doi:10.3390/nano11112796)
Supplement: Supplementary file 1 [file nanomaterials-11-02796-s001.zip › nanomaterials-1416984-supplementary.pdf]

nanomaterials

Supplementary Materials

Poly(Lactic Acid)/Graphite Nanoplatelet Nanocomposite Filaments for Ligament Scaffolds

Magda Silva [1,2,3], Carina Gomes [3], Isabel Pinho [3], Hugo Gonçalves [3], Ana C. Vale [1,2], José A. Covas [3], Natália M. Alves [1,2,*] and Maria C. Paiva [3,*]

1 3B's Research Group, I3Bs—Research Institute on Biomaterials, Biodegradables and Biomimetics, University of Minho Headquarters of the European Institute of Excellence on Tissue Engineering and Regenerative Medicine, Avepark, Barco, 4805-017 Guimarães, Portugal; magda.sof.g.silva@gmail.com (M.S.); cat.cvale@gmail.com (A.C.V.); nalves@i3bs.uminho.pt (N.M.A.)

2 ICVS/3B's, Associate PT Government Laboratory, 4710-057 Braga/4805-017 Guimarães, Portugal

3 Department of Polymer Engineering, Institute for Polymers and Composites, University of Minho, 4800-058 Guimarães, Portugal; carinagomes.96@hotmail.com (C.G.); pg38948@alunos.uminho.pt (I.P.); hugosousagoncalves@protonmail.com (H.G.); jcovas@dep.uminho.pt (J.A.C.)

* Correspondence: nalves@i3bs.uminho.pt (N.M.A.); mcpaiva@dep.uminho.pt (M.C.P.)

1. Supplementary Information

1.1. Production of filaments

The operating parameters used for the production of the filaments Fil3D and FilText are presented in Table S1.

Table S1. Operating parameters used for the production of composite filaments.

Filament		Fil3D				FilText			
		Barrel 1/Barrel 2/Die Temperature(°C)	Screw speed (rpm)	Roll Speed (mm.s^{-1})		Barrel 1/Barrel 2/Die Temperature(°C)	Screw speed (rpm)	Roll Speed (mm.s^{-1})	
				Roll1	Roll2			Roll1	Roll2
**PLA**		130/170/160	45	22.8	25.6	130/170/160	15	232.2	233.6
**PLA + 0.25**	EG	130/170/169	36	24.2	25.6	130/170/160	15	231.5	234.3
**PLA + 0.5**		130/170/169	36	24.9	27.6		15	232.2	254.3
**PLA + 1**		135/170/155	45	31.1	33.2		25	228.8	282.7
**PLA + 2**		135/170/155	65	30.4	32.5		45	228.8	304.8
**PLA + 0.25**	f-EG	123/170/160	45	24.9	28.3	123/170/160	25	268.9	270.2
**PLA + 0.5**		123/170/160	45	25.6	27.6	123/170/160	25	270.9	272.3
**PLA + 1**		135/170/155	65	31.1	34.6	130/170/160	45	246.7	297.2
**PLA + 2**		135/170/155	65	30.4	32.5	130/170/160	65	228.8	309.6
**PLA + 0.25**	(f-EG)+Ag	123/170/160	45	25.6	27.6	130/170/160	25	271.6	273.0
**PLA + 0.5**		123/170/160	45	25.6	29.7		25	271.6	273.7
**PLA + 1**		125/170/155	65	31.1	34.6		45	246.7	302.7
**PLA + 2**		125/170/155	80	29.7	31.8		65	228.8	312.4

1.2. Characterization of functionalized graphite-EDS

EDS tests were performed for [(f-EG)+Ag] to confirm the presence of silver. Figure S1 presents a backscattered electron image of [(f-EG)+Ag] with two different zones (Z1 and Z2) analyzed through EDS and the Ag signal indicates the presence of silver in this graphite, as expected. The C signal is due to graphite, the O signal may be due to residual oxidation of the pristine EG and also to the presence of some benzyl carbamate and the Ag signal indicates the presence of silver in EG, as expected.

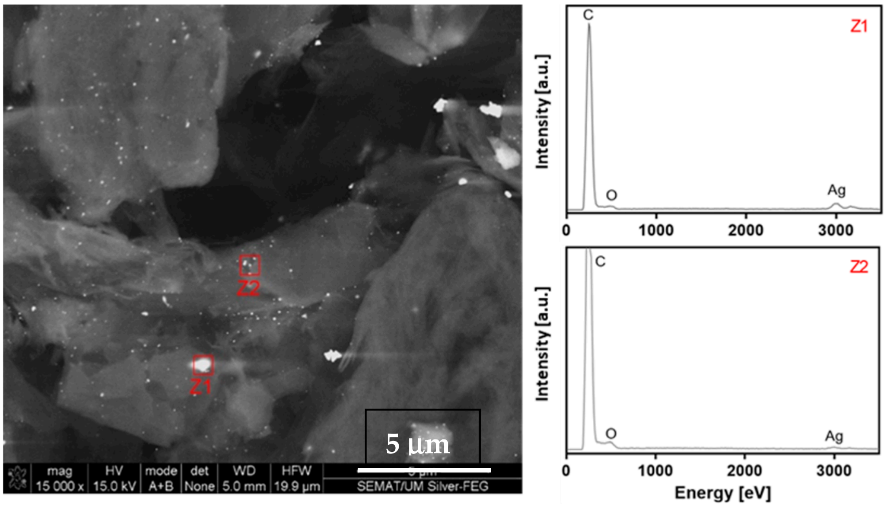

Figure S1. SEM image and EDS analysis of [(f-EG) + Ag].

1.3. Characterization of the composite filaments-SEM

The images of the cross sections of composite filaments reinforced with EG, f-EG and [(f-EG)+Ag] are displayed in Figures S2 and S3.

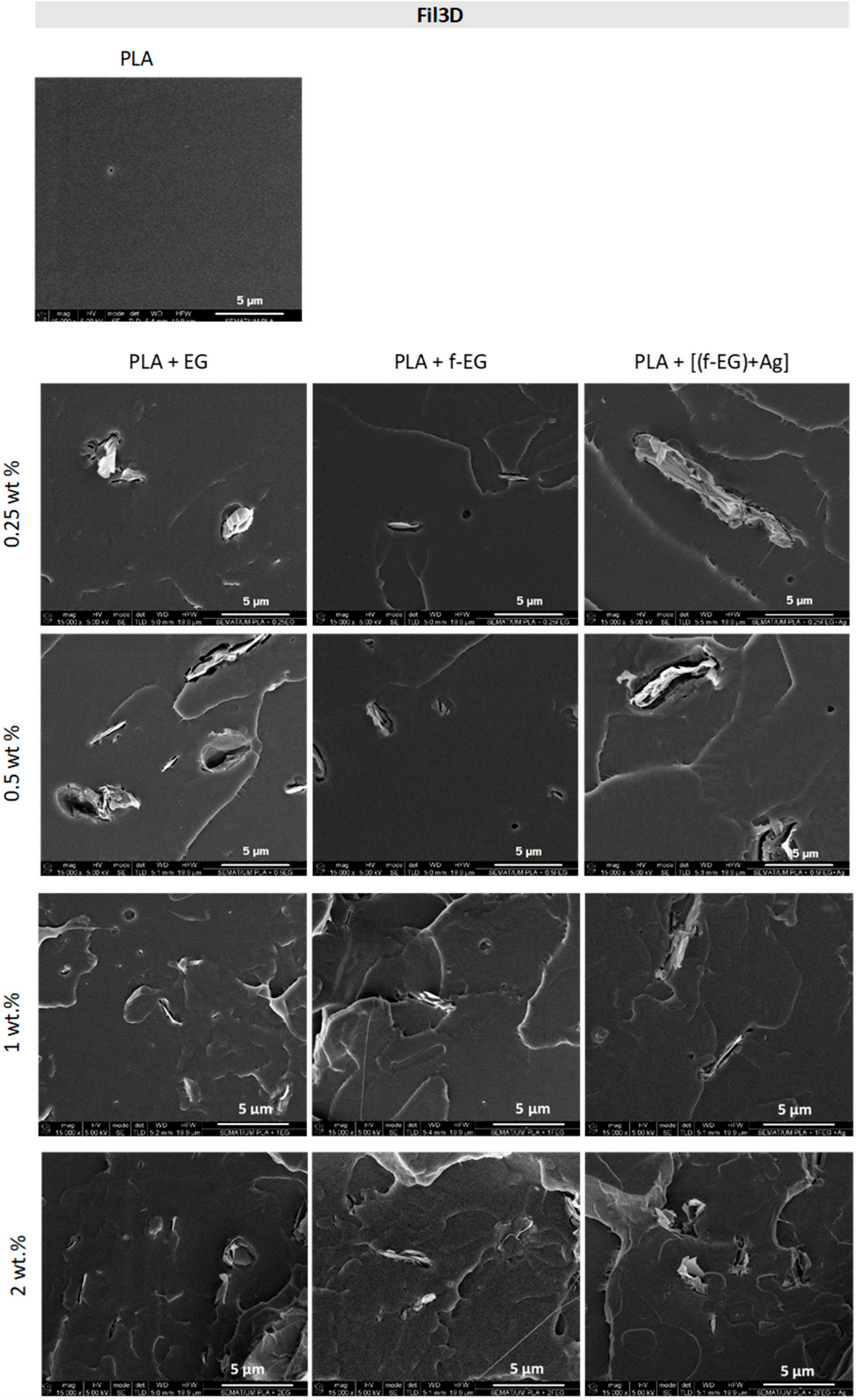

Figure S2. SEM images of Fil3D: PLA; PLA reinforced with 0.25, 0.5, 1 and 2 wt.% of EG, f-EG and [(f-EG)+Ag].

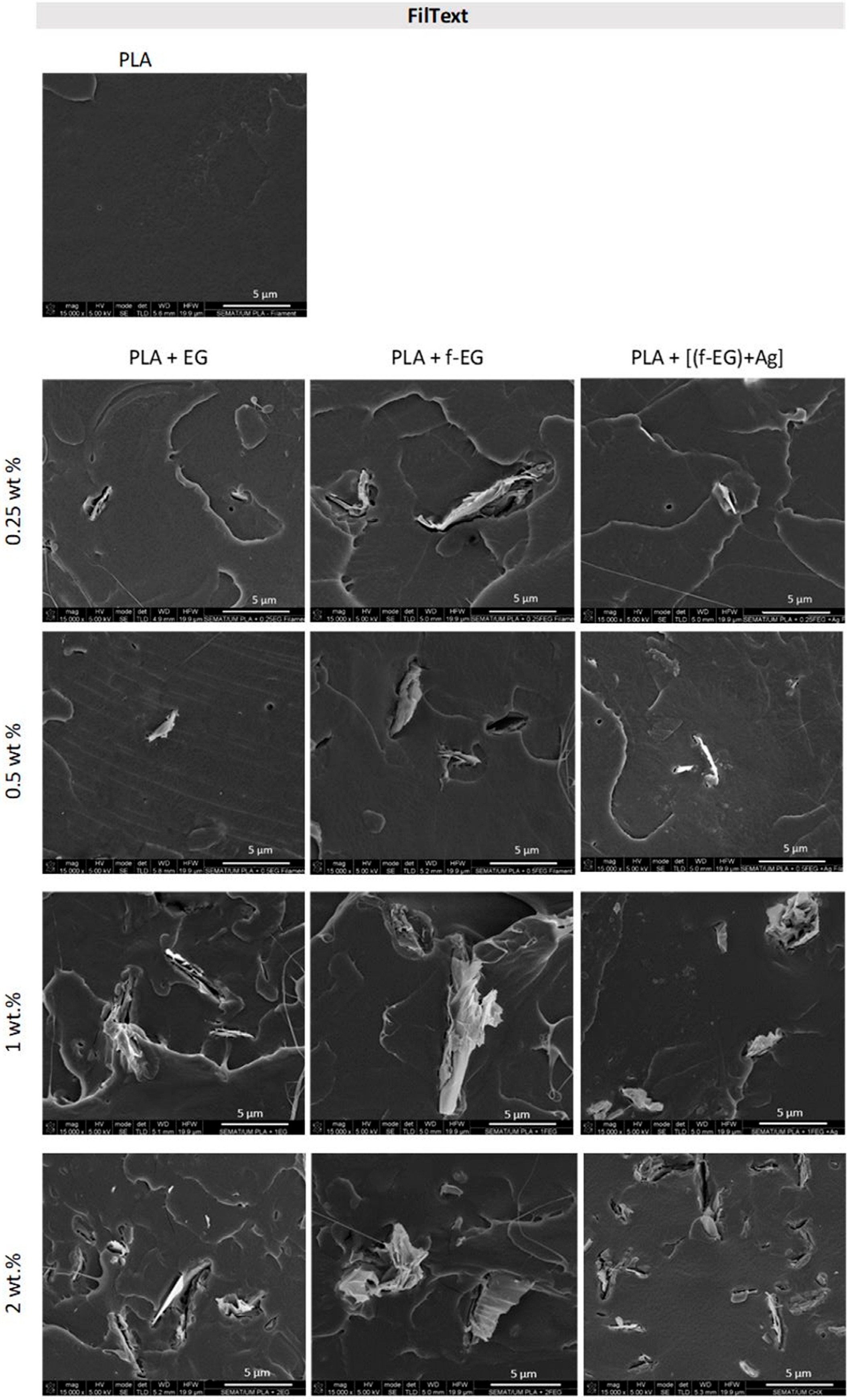

Figure S3. SEM images of FilText: PLA; PLA reinforced with 0.25, 0.5, 1 and 2 wt.% of EG, f-EG and [(f-EG)+Ag].

1.4. Characterization of the composite filaments-EDS

EDS tests were performed and confirmed the presence of Ag nanoparticles in Fil3D and FilText reinforced with [(f-EG)+Ag]-see Figure S4.

The SEM images revealed that the polymeric matrix was degraded by the electron beam used to obtain backscattered electron images, however this was necessary to see the silver particles that appear brighter in the image. The C signal is due to the graphite and the polymeric matrix, the O signal may appear mainly due to the presence of PLA, gold (Au) signal refers to the deposition that was made in the sample for the SEM analysis and, finally, the Ag signal indicates the presence of silver in the composite filaments, as expected.

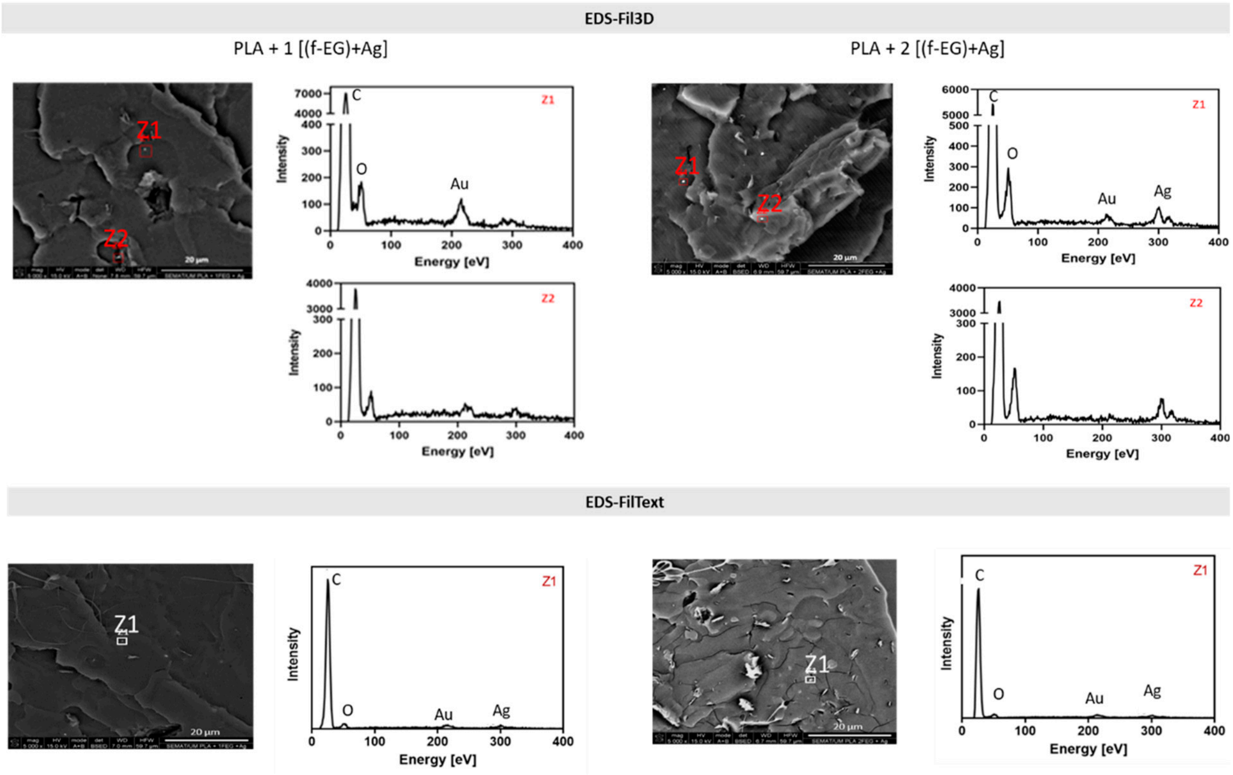

Figure S4. SEM images and EDS analysis of Fil3D and FilText reinforced with 1 and 2wt.% of [(f-EG)+Ag].

1.5. Characterization of the composite filaments-OM

OM images of the composite filament cross sections with different loads of EG, f-EG and [(f-EG)+Ag] are displayed in Figures S5 and S6.

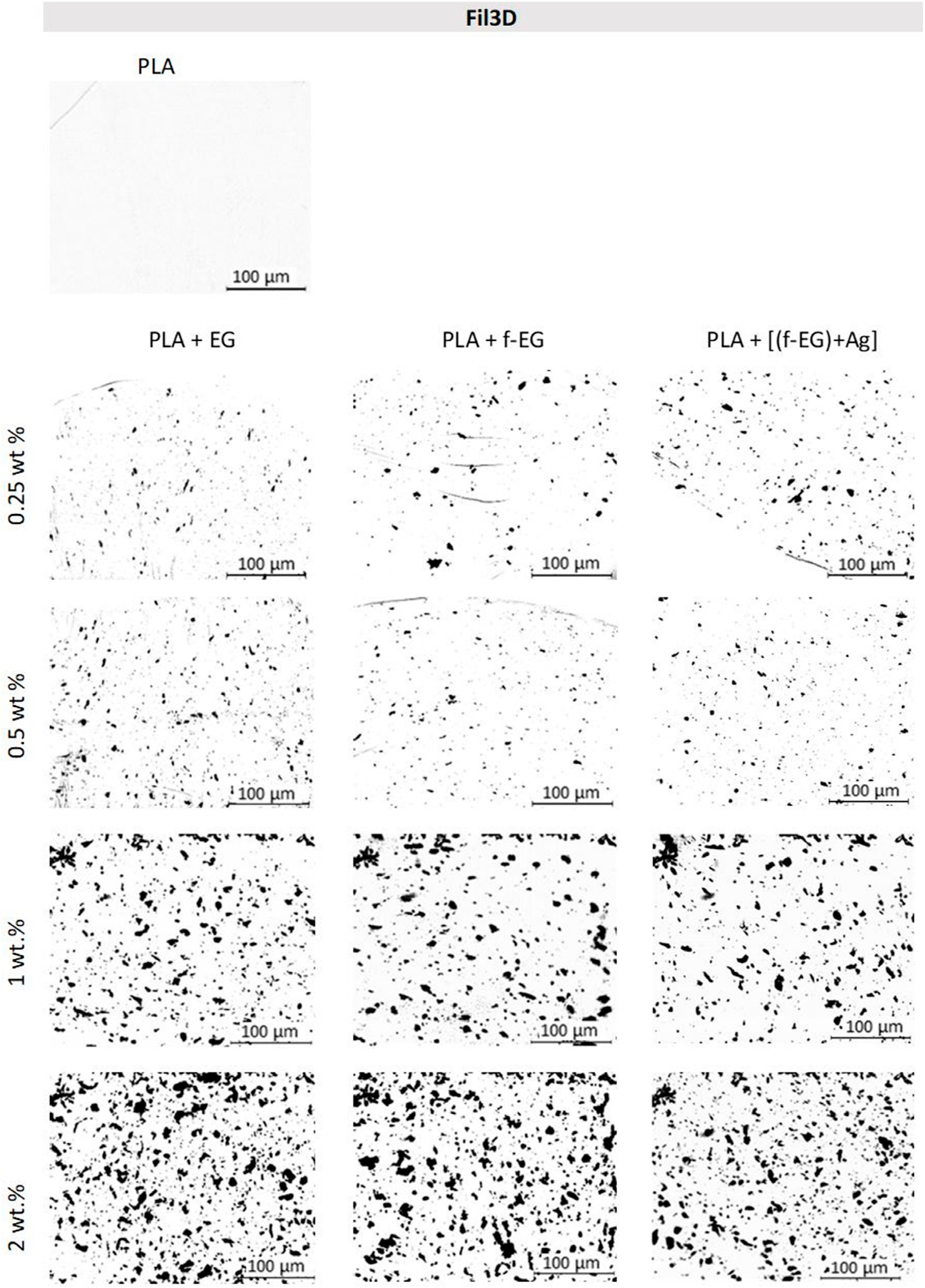

Figure S5. Optical microscopy images of the Fil3D's cross-section, namely PLA; PLA reinforced with 0.25, 0.5, 1 and 2 wt.% of EG, f-EG and [(f-EG)+Ag].

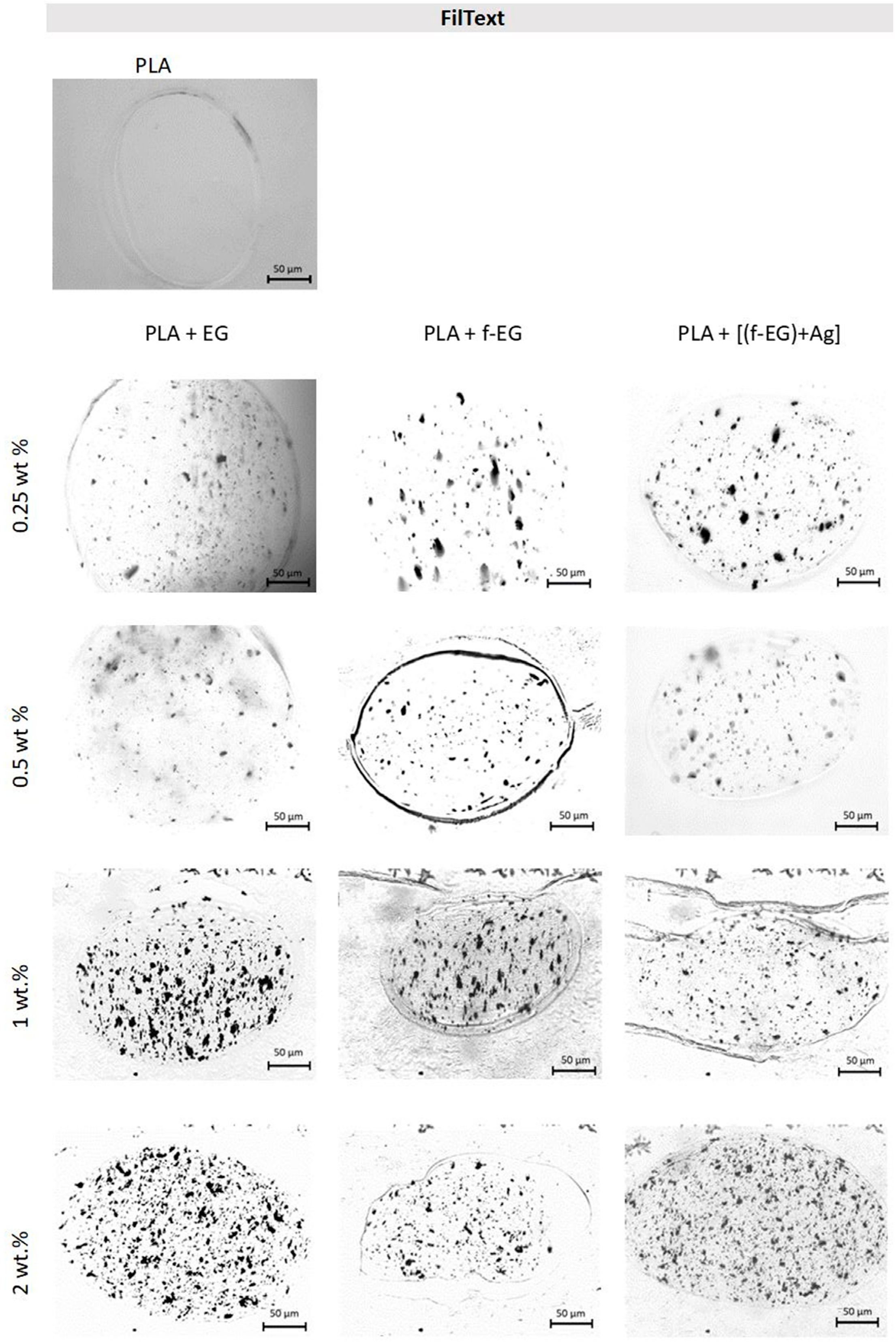

Figure S6. Optical microscopy images of the FilText's cross-section, namely PLA; PLA reinforced with 0.25, 0.5, 1 and 2 wt.% of EG, f-EG and [(f-EG)+Ag].

1.6. Characterization of the composite filaments-DSC

The relevant thermal characteristics T_g, , T_c, T_m, ΔH_m, ΔH_c, and χ_c, of each composition obtained for the first and second heating scans are reported in Tables S2 and S3, respectively.

Table S2. Summary of the thermal properties of Fil3D and FilText obtained for the 1st heating.

Filament		T_g (°C)		T_c (°C)		T_m (°C)		ΔH_c (J/g)		ΔH_m (J/g)		χ_c (%)	
		Fil3D	FilText	Fil3D	FilText	Fil3D	FilText	Fil3D	FilText	Fil3D	FilText	Fil3D	FilText
PLA		57.5	59.2	110.2	104.1	161.9	159.6	30.1	28.9	33.6	29.7	3.7	0.9
PLA + 0.25	EG	57.7	59.8	108.9	102.8	161.7	158.8	29.4	28.3	31.6	29.7	2.4	1.5
PLA + 0.5	EG	58.3	59.4	109.3	104.2	161.7	158.8	28.6	27.0	30.8	28.1	2.4	1.2
PLA + 1	EG	54.7	61.9	117.7	111.3	162.5	160.7	30.0	30.8	35.8	31.4	6.3	0.6
PLA + 2	EG	55.8	55.7	103.6	107.2	161.6	160.7	26.0	30.4	31.6	30.6	6.1	0.2
PLA + 0.25	f-EG	58.3	59.5	109.2	103.4	161.7	158.6	29.4	27.6	32.0	28.6	2.7	1.1
PLA + 0.5	f-EG	58.2	59.2	108.4	105.3	161.9	159.2	28.2	30.4	31.2	31.0	2.4	0.7
PLA + 1	f-EG	55.2	56.3	112.2	110.1	162.2	161.0	28.2	28.3	32.9	28.7	6.3	0.4
PLA + 2	f-EG	56.4	57.0	105.0	107.8	161.9	161.1	27.1	28.8	35.5	29.0	6.1	0.2
PLA + 0.25	(f-EG)+Ag	58.9	59.8	108.9	103.6	161.7	159.2	29.7	25.5	32.7	26.9	3.3	1.5
PLA + 0.5	(f-EG)+Ag	59.4	59.7	109.4	104.1	161.8	159.6	30.4	25.4	32.5	26.8	2.4	1.5
PLA + 1	(f-EG)+Ag	55.6	54.6	108.1	112.1	162.3	161.0	29.3	29.1	33.7	29.5	6.3	0.5
PLA + 2	(f-EG)+Ag	57.1	55.2	107.5	107.5	161.6	160.8	27.2	26.5	34.5	26.8	6.1	0.3

Table S3. Summary of the thermal properties of Fil3D and FilText obtained for the 2nd heating.

Filament		T_g (°C)		T_c (°C)		T_m (°C)		ΔH_c (J/g)		ΔH_m (J/g)		χ_c (%)	
		Fil3D	FilText	Fil3D	FilText	Fil3D	FilText	Fil3D	FilText	Fil3D	FilText	Fil3D	FilText
PLA		60.2	58.5	113	105	161	160	31.9	30.2	34.7	31.5	3.0	1.4
PLA + 0.25	EG	60.7	58.8	117	112	158	159	31.2	30.7	33.7	31.7	2.6	1.1
PLA + 0.5	EG	60.7	58.4	118	113	155	159	28.0	30.7	32.5	31.4	4.7	0.8
PLA + 1	EG	59.0	58.7	116	112	161	161	30.3	29.3	37.5	31.6	7.8	2.6
PLA + 2	EG	59.0	52.0	121	117	156	161	29.3	18.3	36.0	31.6	7.3	14.5
PLA + 0.25	f-EG	60.8	58.9	121	117	156	157	29.8	28.7	34.1	31.0	4.7	2.4
PLA + 0.5	f-EG	60.9	59.1	119	118	156	156	29.3	32.6	32.9	33.6	3.9	1.1
PLA + 1	f-EG	59.0	59.1	118	114	161	161	29.9	26.2	35.6	28.6	6.3	2.6
PLA + 2	f-EG	59.0	58.8	116	111	161	161	33.0	19.4	35.9	28.9	3.2	10.3
PLA + 0.25	(f-EG)+Ag	60.7	60.1	124	118	157	156	28.9	30.6	31.6	32.1	3.0	1.7
PLA + 0.5	(f-EG)+Ag	60.7	59.6	123	119	157	154	30.9	26.7	32.3	30.5	1.5	4.1
PLA + 1	(f-EG)+Ag	59.0	60.4	117	116	161	161	33.1	23.4	35.7	30.0	2.8	7.1
PLA + 2	(f-EG)+Ag	59.2	58.8	115	113	161	161	31.1	18.9	35.8	26.5	5.1	8.3

1.7. Characterization of the composite filaments-biodegradation

PLA and all the composite filaments were immersed in a PBS solution at 37 °C, during 7, 14, 21 and 28 days. The obtained results are detailed in Table S4.

Table S4. Weight loss (%) of Fil3D and FilText.

		Weight loss (%)							
		0-7days		0-14 days		0-21 days		0-28 days	
Filament		Fil3D	FilText	Fil3D	FilText	Fil3D	FilText	Fil3D	FilText
PLA		0.00	0.00	0.00	0.00	0.00	0.00	0.00	0.00
PLA+0.25	EG	0.00	0.00	0.00	0.00	0.00	0.00	0.00	0.00
PLA+0.5	EG	0.00	0.00	0.00	0.00	0.00	0.00	1.39	0.00
PLA+0.25	f-EG	0.00	0.00	0.00	0.00	0.00	0.00	0.00	0.00
PLA+0.5	f-EG	0.00	0.00	1.37	0.00	0.00	0.00	0.00	0.00
PLA+0.25	f-EG+Ag	0.00	0.00	1.32	0.00	0.00	0.00	0.00	0.00
PLA+0.5	f-EG+Ag	0.00	0.00	1.24	0.00	0.00	0.00	0.00	0.00
